# Effects of glucose on lactose synthesis in mammary epithelial cells from dairy cow

**DOI:** 10.1186/s12917-016-0704-x

**Published:** 2016-05-26

**Authors:** Ye Lin, Xiaoxu Sun, Xiaoming Hou, Bo Qu, Xuejun Gao, Qingzhang Li

**Affiliations:** Key Laboratory of Dairy Science of Education Ministry, Northeast Agricultural University, Harbin, 150030 China

**Keywords:** Dairy cow, Mammary epithelial cell, Glucose, Lactose synthesis, AKT1

## Abstract

**Background:**

Lactose, as the primary osmotic component in milk, is the major determinant of milk volume. Glucose is the primary precursor of lactose. However, the effect of glucose on lactose synthesis in dairy cow mammary glands and the mechanism governing this process are poorly understood.

**Results:**

Here we showed that glucose has the ability to induce lactose synthesis in dairy cow mammary epithelial cells, as well as increase cell viability and proliferation. A concentration of 12 m*M* glucose was the optimum concentration to induce cell growth and lactose synthesis in cultured dairy cow mammary epithelial cells. In vitro, 12 m*M* glucose enhanced lactose content, along with the expression of genes involved in glucose transportation and the lactose biosynthesis pathway, including GLUT1, SLC35A2, SLC35B1, HK2, β4GalT-I, and AKT1. In addition, we found that *AKT1* knockdown inhibited cell growth and lactose synthesis as well as expression of GLUT1, SLC35A2, SLC35B1, HK2, and β4GalT-I.

**Conclusions:**

Glucose induces cell growth and lactose synthesis in dairy cow mammary epithelial cells. Protein kinase B alpha acts as a regulator of metabolism in dairy cow mammary gland to mediate the effects of glucose on lactose synthesis.

**Electronic supplementary material:**

The online version of this article (doi:10.1186/s12917-016-0704-x) contains supplementary material, which is available to authorized users.

## Background

The basic function of the lactating mammary gland is to produce milk, providing nutrients for growth and development of the offspring. The main constituents of bovine milk are lactose, proteins, and lipids [1]. Among these, lactose is the major carbohydrate of milk. It plays a primary role in milk production because it represents the main osmotic constituent in milk and draws water into the mammary epithelial cells [2].

Lactose is synthesized from free glucose and uridine diphosphate (UDP)-galactose. In lactating ruminants, the mammary gland can consume up to 85 % of circulating glucose [3]. Uridine diphosphate-galactose is also derived from glucose. Analysis of lactose synthesis in rodent and bovine models of secretory activation has demonstrated increases in a number of enzyme activities and carbohydrate metabolites that together form the fundamental framework of the lactose synthesis pathway [4, 5]. The lactose synthesis reaction is catalyzed by lactose synthase (UDP-galactose : D-glucose l-galactosyl transferase, EC 2.4.1.22), a complex of β-1, 4-galactosyltransferase (β4GalT-I) and the essential cofactor α-lactalbumin (α-LA), in the Golgi compartment [6, 7]. The expression of α-LA is very low during pregnancy and increases significantly with parturition [8]. Beginning in late pregnancy, the β4GalT-I level in the mammary gland is estimated to increase about 50-fold in preparation for lactose biosynthesis [8]. As the substrate of lactose synthase, the glucose concentration in the lactating mammary epithelial cells is higher than usual [1]. Generally, animal nutrition has a substantial effect on milk composition [9]. Glucose is a dietary factor that affects milk synthesis [10], but whether glucose supplementation can affect lactose synthesis in dairy cow mammary gland is not well understood.

Lactose synthesis and secretion by the mammary gland involve the expression of a large number of genes [9]. However, few studies have focused on the effects of glucose supplementation on the expression of genes involved in lactose biosynthesis. The protein kinase B (PKB, also known as AKT) is a critical downstream effector in multiple signal transduction pathways and regulates cellular proliferation, survival, and metabolism [11]. Among the members of the AKT family, only AKT1 is upregulated in the mammary gland during pregnancy and lactation [12]. Knockout studies have demonstrated that AKT1 has roles in the functional differentiation of the secretory epithelium and in metabolic pathways that regulate milk synthesis [13]. Loss of AKT1 results in failure of the coordinated metabolic responses required for the establishment of lactation at parturition, including increased glucose uptake and lipid synthesis, which in turn results in decreased milk production [13]. The lactation defect observed in *AKT1*^–/–^ mice is thus due to a metabolic defect that results from a failure to upregulate glucose transporter 1 (GLUT1) and other AKT1-specific target genes [11]. It is unknown whether glucose supplementation modulation of lactose synthesis could be related to *AKT1* expression in dairy cow mammary gland.

In this study, we hypothesized glucose supplementation could affect lactose synthesis in lactating mammary gland of dairy cow. Additionally, glucose induced lactose synthesis is related to AKT1 expression in lactating cow mammary epithelial cells. To meet these objectives, we evaluated the effects of different concentrations of glucose on mammary epithelial cell survival, proliferation, and lactose synthesis. The expression of genes known to be involved in glucose transportation and lactose synthesis was examined by quantitative real-time PCR (qPCR) and western blot when cells were cultured with DMEM containing 12 m*M* glucose. To evaluate if glucose modulates lactose synthesis via AKT1 activation, siRNA-mediated knockdown of *AKT1* in cultured mammary epithelia cells was performed.

## Results

### Effect of glucose on lactose synthesis in dairy cow mammary epithelial cells

**Fig. 1:**
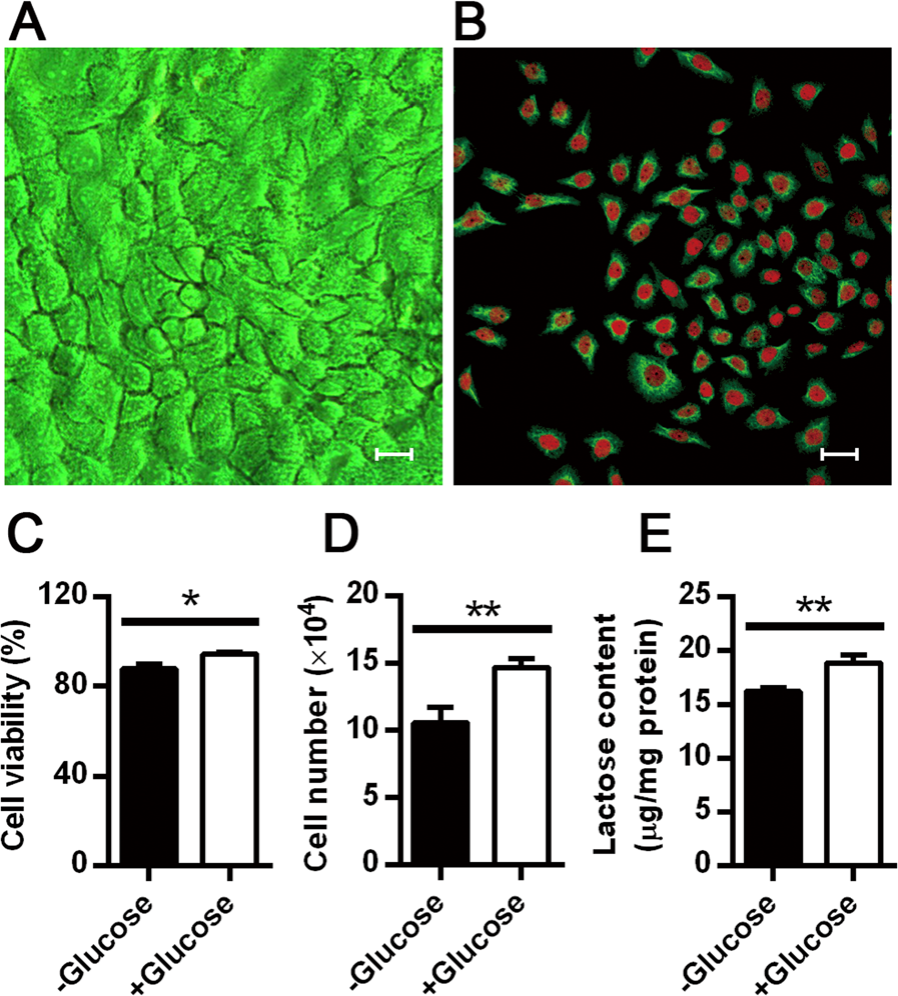
Glucose induces dairy cow mammary epithelial cell growth and lactose synthesis. **a** Mammary epithelial cells isolated from mid-lactating mammary tissues of dairy cows were acquired using a phase-contrast light microscopy with a Leica L 40 × 0.5 PH2 objective. **b** Immunofluorescence staining for cytokeratin 18 in dairy cow mammary epithelial cells was acquired using a confocal microscopy with a Leica HCX PL Apo CS 40 × 1.25 oil objective. Cytokeratin 18 was stained with FITC (green), and nuclei were stained with propidium iodide (red). For A and B, Scale bar, 75 μm. **c**, **d** The effect of glucose on cell viability (**c**) and proliferation (**d**) in dairy cow mammary epithelial cells. **e** Lactose secretion from dairy cow mammary epithelial cells cultured with or without glucose. Lactose content in medium was measured with the Lactose/d-Glucose (Rapid) Assay kit (K-LACGAR, Megazyme, Ireland, UK). For **c**, **d**, and **e**, mammary epithelial cells were cultured in DMEM with high glucose (+Glucose, 25 mM) or without glucose (-Glucose) for 24 h. Data are shown as the mean ± SEM from 3 independent replicates. *P < 0.05, **P < 0.01

Glucose is the primary precursor of lactose in lactating mammary glands. In animal and human models, plasma glucose gives rise to the vast majority of the monosaccharides of lactose [14, 15]. To investigate if glucose supplementation has the ability to induce lactose synthesis in lactating dairy cow mammary gland, we generated mammary epithelial cells from mid-lactating mammary tissues of dairy cows (Fig. 1a). Immunofluorescence staining of cytokeratin 18 was observed in the cytoplasm (Fig. 1b), indicating that the cells we cultured were purified mammary epithelial cells [16].

To detect the effects of glucose on mammary epithelial cell survival, proliferation, and lactose biosynthesis, mammary epithelial cells were cultured in DMEM with or without glucose for 24 h. Cell viability (Fig. 1c) and proliferation (Fig. 1d) were both markedly increased in mammary epithelial cells cultured with high glucose compared with those in glucose-free medium (*P* < 0.05). We also measured the lactose secreted from mammary epithelial cells into the medium. The amount of lactose in medium collected from cells cultured with high glucose for 24 h was significantly higher than that from glucose free-cultured cells (*P* < 0.01, Fig. 1e). These data suggested that glucose induced mammary epithelial cell growth and lactose biosynthesis.

**Fig. 2:**
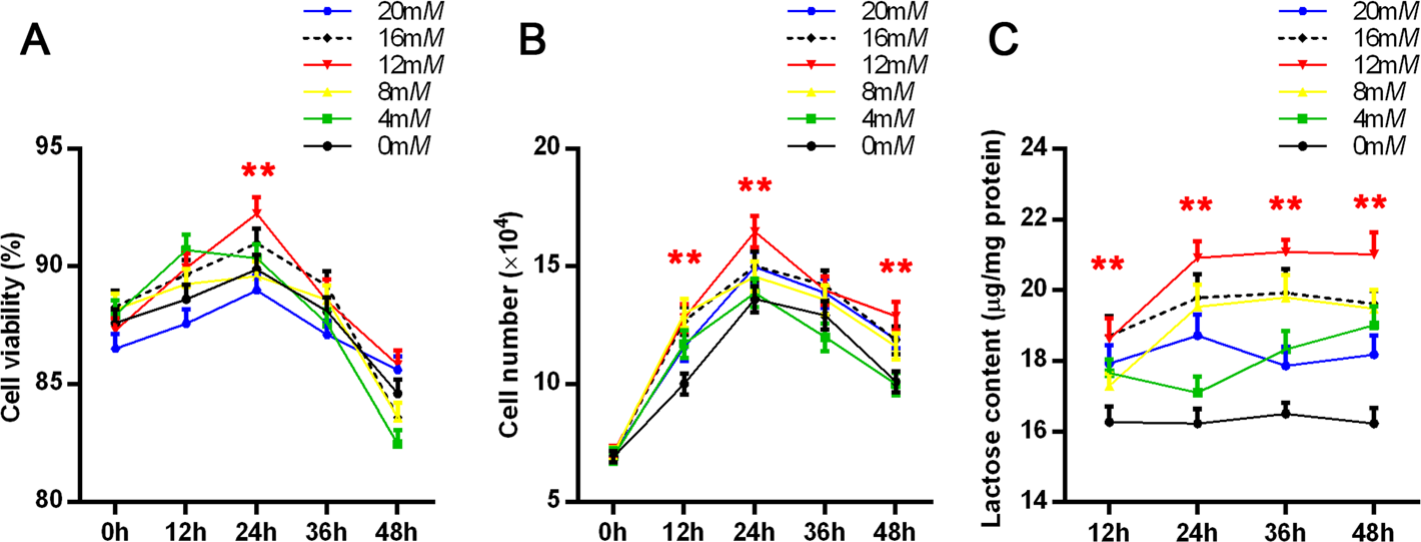
The effect of glucose concentration on cell growth and lactose synthesis. **a**, **b** The effect of glucose concentration on cell viability (**a**) and proliferation (**b**) in dairy cow mammary epithelial cells. **c** The effect of glucose concentration on lactose synthesis in dairy cow mammary epithelial cells. Dairy cow mammary epithelial cells were cultured in DMEM with glucose at concentrations ranging from 0 to 20 m*M*. The effects of glucose concentration on cell viability, proliferation, and lactose content were examined after 12, 24, 36, and 48 h of treatment. Data are shown as the mean ± SEM from 3 independent replicates. *P < 0.05; **P < 0.01 versus corresponding value for cells cultured in DMEM with 0 m*M* glucose

Next, we measured the effects of different concentrations of glucose on lactose synthesis in dairy cow mammary epithelial cells. Mammary epithelial cells were cultured in DMEM with glucose at concentrations ranging from 0 to 20 m*M*. The effects of glucose concentration on cell viability and proliferation were examined after 12, 24, 36, and 48 h of treatment. Cell viability and proliferation both increased for the first 24 h, followed by a decrease (Fig. 2a, b). However, the viability and proliferation of only those cells cultured with 12 m*M* glucose were upregulated significantly, peaking at 24 h, compared with the other glucose concentration groups (at 24 h). The lactose content in the medium increased for the first 24 h, followed by a plateau when cells were cultured with 8, 12, and 16 m*M* glucose. Similarly, lactose content reached highest in the medium when cells were cultured with 12 m*M* glucose for the first 24 h (Fig. 2c). As a result, a concentration of 12 m*M* glucose was determined to be the optimum concentration to induce lactose synthesis in cultured dairy cow mammary epithelial cells.

### Effect of glucose on expression of genes involved in lactose synthesis in dairy cow mammary epithelial cells

**Fig. 3:**
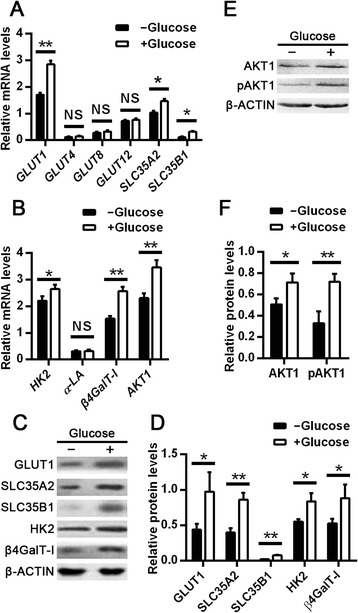
Effect of glucose on expression of genes involved in lactose synthesis. **a** Quantitative real-time PCR analysis of the mRNAs encoding glucose and UDP-galactose transporters, including glucose transporter 1 (GLUT1), glucose transporter 4 (GLUT4), glucose transporter 8 (GLUT8), glucose transporter 12 (GLUT12), solute carrier family 35 member A2 (SLC35A2), and solute carrier family 35 member B1 (SLC35B1). **b** Quantitative real-time PCR analysis of the mRNAs encoding key enzymes and regulators in the lactose synthesis pathway, including hexokinase-2 (HK2), α-lactalbumin (α-LA), β-1,4-galactosyltransferase 1 (β4GalT-I), and protein kinase B alpha (AKT1). For **a** and **b**, total RNA was isolated from dairy cow mammary epithelial cells treated with or without 12 m*M* glucose for 24 h. The mRNA levels were measured by qPCR and normalized to *β-ACTIN* mRNA. **c**, **d** Western blot analysis (**c**) and quantification (**d**) of proteins involved in lactose synthesis. **e**, **f** Western blot analysis (**e**) and quantification (**f**) of AKT1 and pAKT1. For **c** and **e**, protein lysates from dairy cow mammary epithelial cells treated with or without 12 m*M* glucose for 24 h were collected for western blot analysis. Data are shown as the mean ± SEM from 3 independent replicates. *P < 0.05; **P < 0.01; NS, no statistical significance

In lactating mammary gland, lactose synthase catalyzes the conversion of glucose and UDP-galactose to lactose in the Golgi [8]. Glucose is passed across the plasma membrane and Golgi membrane into the Golgi lumen by GLUTs [17]. Uridine diphosphate-galactose is actively transported into the Golgi lumen by solute carrier family 35 member A2 (SLC35A2) and solute carrier family 35 member B1 (SLC35B1) [18]. To explore the molecular process by which glucose induces lactose synthesis, we first examined the expression of *GLUT1*, *GLUT4, GLUT8, GLUT12*, *SLC35A2*, and *SLC35B1,* which mediate glucose and UDP-galactose transportation in mammary gland [19–21]. As shown in Fig. 3a, *GLUT1, SLC35A2*, and *SLC35B1* mRNA levels were significantly increased in mammary epithelial cells cultured in DMEM with 12 m*M* glucose for 24 h (*P* < 0.05), whereas *GLUT4, GLUT8,* and *GLUT12* mRNA levels did not change compared to the control. Next, we examined the expression of hexokinase 2 (*HK2*), *α-LA*, *β4GalT-I*, and *AKT1*, which are involved in catalyzing and regulating lactose biosynthesis in lactating mammary gland [22]. The results showed that *HK2*, *β4GalT-I*, and *AKT1* mRNA levels were significantly increased in mammary epithelial cells cultured in DMEM with 12 m*M* glucose for 24 h (*P* < 0.05), whereas expression of *α-LA* remained relatively unchanged (Fig. 3b).

On the basis of the mRNA expression data, we analyzed protein expression of GLUT1, SLC35A2, SLC35B1, HK2, β4GalT-I, AKT1, and pAKT1 in mammary epithelial cells by western blot. Compared with the control, the levels of GLUT1, SLC35A2, and SLC35B1 were significantly increased (*P* < 0.05) in mammary epithelial cells cultured in DMEM with 12 m*M* glucose for 24 h. Consistent with this, HK2 and β4GalT-I levels were also increased (*P* < 0.05) (Fig. 3c, d). Protein kinase B alpha and its active form, pAKT1, were also upregulated in mammary epithelial cells cultured in DMEM with 12 m*M* glucose compared with the control (*P* < 0.05; Fig. 3e, f). Overall, these results strongly suggested that glucose induced lactose synthesis in mammary epithelial cells by upregulating GLUT1, SLC35A2, SLC35B1, HK2, β4GalT-I, and AKT1.

### Relationship between AKT1 and glucose modulation of lactose synthesis in dairy cow mammary gland

**Fig. 4:**
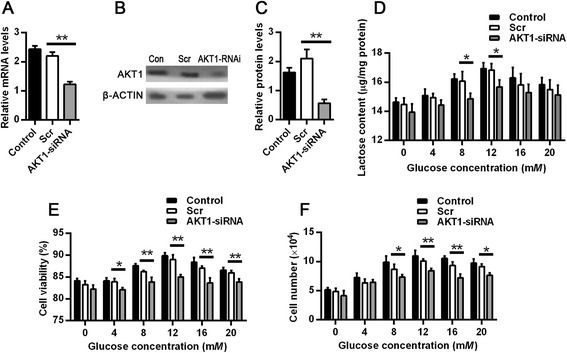
Glucose modulates cell growth and lactose synthesis via AKT1 activation. Nonspecific scrambled siRNA (Scr) was used as a negative control in all experiments. **a** Relative *AKT1* mRNA levels in siRNA-transfected dairy cow mammary epithelial cells. The mRNA levels were measured by qPCR and normalized to *β-ACTIN* mRNA. **b**, **c** Western blot analysis (**b**) and quantification (**c**) of AKT1 in siRNA-transfected dairy cow mammary epithelial cells. The protein levels were normalized to β-ACTIN. **d**, **e**, **f** The effect of glucose concentration on lactose synthesis (**d**), cell viability (**e**), and proliferation (**f**) in dairy cow mammary epithelial cells when cells were transiently transfected with *AKT1* siRNA. siRNA-transfected dairy cow mammary epithelial cells were cultured in DMEM with glucose at concentrations ranging from 0 to 20 m*M* for 24 h. The effect of glucose concentration on lactose content, cell viability, and proliferation were examined. Data are shown as the mean ± SEM from 3 independent replicates. *P < 0.05; **P < 0.01

Next we examined the regulatory mechanism underlying the effects of glucose on lactose synthesis. Protein kinase B alpha has a critical role in the proliferation, survival, and metabolism of numerous cell types [23, 24]. The above findings demonstrated that AKT1 and pAKT1 expression were both upregulated by glucose in vitro cow mammary epithelial cells. To determine whether glucose modulates lactose synthesis via AKT1 activation in dairy cow mammary gland, siRNA-mediated knockdown of *AKT1* in dairy cow mammary epithelial cells was performed, followed by measurement of *AKT1* mRNA and protein 48 h later. The expression of *AKT1* mRNA was suppressed significantly by transfection with *AKT1* siRNA (*P* < 0.01) but not by the scrambled siRNA (Fig. 4a). Consistent with the qPCR results, knockdown of *AKT1* in mammary epithelial cells also led to substantially lower levels of AKT1 (*P* < 0.01; Fig. 4b, c).

We then investigated the effect of *AKT1* knockdown on lactose synthesis in mammary epithelial cells and measured lactose production under both basal (without glucose) and stimulated (glucose concentrations ranging from 4 to 20 m*M*) conditions. Compared with the control, *AKT1* knockdown decreased lactose synthesis in both basal and glucose-stimulated conditions. The effect at 0 m*M* (basal) was not significant, and the inhibition effect was significant at 8 *mM* and 12 m*M* glucose (Fig. 4d). We further examined the effect of *AKT1* knockdown on cell viability and proliferation when cells were cultured in medium with different concentrations of glucose (from 0 to 20 m*M*). Compared with the control, cell viability and proliferation were both significantly decreased when *AKT1* was knocked down (Fig. 4e, f). However, cell viability and proliferation were increased by treatment with 12 m*M* glucose compared to other glucose concentrations when *AKT1* was knocked down**.**

**Fig. 5:**
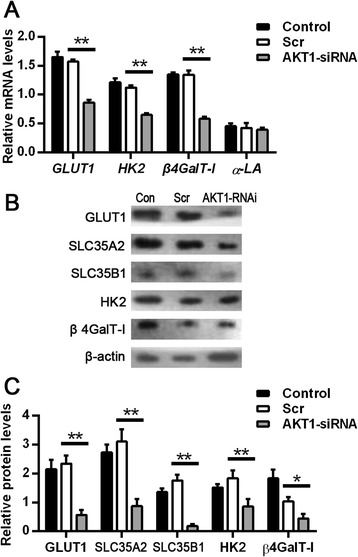
Protein kinase B knockdown decreased the expression of genes involved glucose transportation and lactose synthesis. Nonspecific scrambled siRNA (Scr) was used as a negative control in all experiments. **a** Relative mRNA levels of glucose transporter 1 (GLUT1), hexokinase-2 (HK2)*,* β-1,4-galactosyltransferase 1 (β4GalT-I), and α-lactalbumin (α-LA) in siRNA-transfected dairy cow mammary epithelial cells. siRNA-transfected dairy cow mammary epithelial cells were cultured in DMEM with 12 m*M* glucose for 24 h. Cells were collected and the total RNA was isolated for gene expression analysis. The mRNA levels were measured by qPCR and normalized to *β-ACTIN* mRNA. **b**, **c** Western blot analysis (**b**) and quantification (**c**) of protein expression levels of GLUT1, solute carrier family 35 member A2 (SLC35A2), solute carrier family 35 member B1 (SLC35B1), HK2, and β4GalT-I in siRNA-transfected dairy cow mammary epithelial cells. siRNA-transfected dairy cow mammary epithelial cells were cultured in DMEM with 12 m*M* glucose for 24 h. Cells were collected for western blot analysis, and the protein levels were normalized to β-ACTIN. Data are shown as the mean ± SEM from 3 independent replicates. *P < 0.05; **P < 0.01

We next examined whether *AKT1* knockdown in dairy cow mammary epithelial cells cultured in medium with 12 m*M* glucose downregulated the expression of a glucose transporter and genes encoding enzymes involved in lactose synthesis. Quantitative real-time PCR analysis showed that the mRNA levels of *GLUT1*, *HK2,* and *β4GalT-I* in *AKT1* knockdown mammary epithelial cells were significantly reduced (*P* < 0.01), whereas the expression of *α-LA* remained relatively unchanged (Fig. 5a). Consistent with these qPCR results, knockdown of *AKT1* in mammary epithelial cells also led to significantly lower levels of GLUT1, HK2, and β4GalT-I, as well as SLC35A2 and SLC35B1 (*P* < 0.05; Fig. 5b, c). Overall, these data strongly support the hypothesis that glucose modulation of lactose synthesis is related to *AKT1* expression in dairy cow mammary gland.

## Discussion

Lactose synthesis is related to milk volume. Glucose is the primary precursor of lactose. Once taken up by the lactating mammary epithelial cell, glucose is either used in the synthesis of lactose or processed by glycolysis to provide energy [25]. Compared with non-lactating dairy cows, lactating dairy cows have an approximately 4-fold higher requirement for glucose [26]. Because lactating dairy cows need more glucose than non-lactating dairy cows, one might reasonably expect that glucose availability could affect lactose synthesis in lactating dairy cow mammary gland. In this study, we observed that glucose increased lactose synthesis in cultured dairy cow mammary epithelial cells, as well as cell viability and proliferation. A concentration of 12 m*M* glucose was the optimum concentration to induce lactose synthesis in cultured dairy cow mammary epithelial cells within 24 h. Protein kinase B alpha was activated by glucose in lactose synthesis process. Glucose transporter 1, SLC35A2, SLC35B1, HK2, and β4GalT-I expression were also increased as a result of AKT1 signaling in dairy cow mammary gland.

In mammals, glucose transport across the basolateral membrane of epithelial cells is carried out by glucose transporters [17]. In the current research, we examined the expression of *GLUT1*, *GLUT4*, *GLUT8*, and *GLUT12*, which has been previously reported in mammary gland [19–21]. Glucose induced significant increases in *GLUT1* mRNA and protein levels in dairy cow mammary epithelial cells but did not affect *GLUT4, GLUT8*, and *GLUT12* mRNA, suggesting that GLUT1 is one of the glucose transporters induced by glucose in dairy cow mammary gland for lactose synthesis. Glucose transporter 1 is abundantly expressed in bovine mammary gland and increases during pregnancy and lactation [27]. Our result is in agreement with a report that GLUT1 is the main extracellular and intercellular (Golgi membrane) glucose transporter in mammary epithelial cells [28]. In mouse and dairy goat mammary epithelial cells, GLUT1 is mainly distributed along the plasma membrane and Golgi apparatus [28, 29]. The presence of GLUT1 on the Golgi membrane appears to be specific to mammary epithelial cells, as most cells do not have this glucose transporter on the Golgi membrane [28].

In rat and mouse mammary glands, GLUT4 expression markedly decreases during pregnancy and practically disappears during lactation [30, 31]. This is because GLUT4 is mainly located in the adipose compartment, not in the plasma membrane [31]. In bovine mammary gland, the expression of GLUT8 increases 10-fold from prepartum to postpartum, and fairly strong GLUT8 staining is observed at both the apical and basolateral membrane of lactating bovine mammary epithelial cells [17]. But our results showed that *GLUT8* expression in cells cultured with 12 m*M* glucose did not changed compared to the control (without glucose), which is consistent with the report that *GLUT8* mRNA is not affected by various concentration of glucose in cultured bovine mammary epithelial cells [32]. Although GLUT12 is detected in the apical plasma membrane of epithelial cells during lactation in rats, forced weaning results in decreased cytoplasmic GLUT1 staining intensity but no change in GLUT12 staining [20]. Our data, along with previous evidence, demonstrate that GLUT1 is the main glucose transporter in dairy cow mammary epithelial cells for lactose synthesis, and its levels are regulated by glucose.

The synthesis of lactose takes place in the Golgi compartment of mammary epithelial cells. Lactose synthesis paralleled the induction of expression of proteins involved in UDP-galactose synthesis and its transport across the Golgi membrane. The initial step in the synthesis of UDP-galactose is phosphorylation of glucose by HK. Bovine mammary epithelial cells grown in higher glucose concentrations have higher HK activity [32]. In rodents, HK1 is expressed in the mammary gland throughout pregnancy and lactation, whereas HK2 is expressed only following parturition [33]. In the current study, we detected *HK2* mRNA and protein expression in cultured dairy cow mammary epithelial cells, and this expression was upregulated when cells were treated with 12 m*M* glucose for 24 h. These expression data are positively correlated with lactose synthesis, indicating that *HK2* expression was affected by glucose during lactation. Glucose has the ability to induce *HK2* expression in mammary epithelial cells, but various concentrations of glucose do not elevate *HK2* mRNA expression levels [32]. Uridine diphosphate-galactose is actively transported into the Golgi lumen by SLC35A2 and SLC35B1. We found that the levels of SLC35A2 and SLC35B1 were markedly increased in mammary epithelial cells treated with 12 m*M* glucose for 24 h. Similarly, the expression of SLC35A2 and SLC35B1 strongly correlated with lactose concentration.

In the Golgi compartment, the biosynthesis of lactose involves the combination of free glucose and UDP-galactose, which is catalyzed by the lactose synthase system. This enzyme complex is composed of β4GalT-I and α-LA [7]. β-1, 4-galactosyltransferase is a constitutively expressed, trans-Golgi resident type II membrane-bound glycoprotein [34]. In mammals, β4GalT-I is required for the tissue-specific production of lactose, which takes place exclusively in the lactating mammary gland. α-lactalbumin is a noncatalytic mammalian protein expressed de novo exclusively in the mammary gland during lactation [35, 36]. The physiological function of α-LA is to lower the glucose *K*_m_ of β4GalT-I so that it may be used maximally for the synthesis of lactose and it changes the specificity of β4GalT-I from N-acetylglucosamine to glucose [37]. Mice with a null mutation of the *α-LA* gene fail to lactate [38]. In this study, we found that *β4GalT-I* levels were upregulated in the presence of additional glucose, but the expression of *α-LA* in dairy cow mammary epithelial cells was not affected by glucose. This result is in agreement with the previous report that elevated level of glucose increases *β4GalT-I* expression, but not *α-LA* in bovine mammary epithelial cells [39]. Our data, along with previous evidence, indicate that glucose supplementation may stimulate lactose synthesis partly by altering the expression of *β4GalT-I*, but not *α-LA*.

In this study, we also demonstrate that glucose modulation of lactose synthesis is likely related to AKT1 in lactating mammary glands of dairy cows. Protein kinase B alpha serves as a potent survival factor for secretory epithelial cells [40]. Protein kinase B alpha is upregulated in the mammary gland during pregnancy and lactation, and knockout studies have demonstrated that this particular serine-threonine protein kinase plays a role in the functional differentiation of the secretory epithelium and in metabolic pathways that regulate milk synthesis [41]. In our experiments, *AKT1* knockdown decreased both basal and glucose-stimulated lactose synthesis, as well as cell viability and proliferation, indicating that *AKT1* is required for lactose synthesis in response to glucose.

The protein kinase B family stimulates transport and metabolism of glucose [42]. In hematopoietic cell line, AKT1 activation increases the localization of GLUT1 to the cell surface and maintenance of HK function [43]. In mammary gland, previous studies have shown that mice lacking *AKT1* exhibit a pronounced metabolic defect during late pregnancy and lactation that results from a failure to upregulate GLUT1 [11, 13]. Our data showed that knockdown of *AKT1* in dairy cow mammary epithelial cells decreased both the mRNA and protein expression of *GLUT1* in response to glucose. These data, along with previous evidence, demonstrate that AKT1 is required for upregulation of glucose uptake in dairy cow mammary epithelial cells. Consistent with a role in upregulation of GLUT1, *AKT1* knockdown also resulted in deceased expression of SLC35A2 and SLC35B1, as well as HK2 and β4GalT-I, in dairy cow mammary epithelial cells.

## Conclusions

Glucose supplementation has the ability to induce lactose synthesis in in-vitro bovine mammary epithelial cells. Glucose modulation of lactose synthesis is related to AKT1 activation in lactating mammary glands of dairy cows. Additionally, GLUT1, SLC35A2, SLC35B1, HK2, and β4GalT-I expression were increased as a result of AKT1 signaling in dairy cow mammary gland.

## Methods

### Mammary epithelial cell cultures

Primary mammary epithelial cells were prepared from mid-lactating mammary tissues of dairy cows as described previously [44]. Briefly, lactating cows were slaughtered and several pieces of mammary parenchyma tissue were aseptically removed from the midregion of the mammary glands. Mammary epithelial cells were isolated from mammary tissues by collagenase digestion and cultured in DMEM/F12 (12400–024, Life Technologies, Carlsbad, CA, USA) containing 10 % (v/v) fetal bovine serum (16000–044, Life Technologies, Carlsbad, CA, USA), 100 U/mL penicillin, 100 μg/mL streptomycin, and lactating hormones (5 μg/mL insulin, 1 μg/mL prolactin, and 1 μg/mL hydrocortisone; Sigma-Aldrich, St Louis, MO, USA) on cell culture dishes coated with 0.5 % rat tail collagen. Cells were maintained at 37 °C in an atmosphere containing 5 % CO_2_. When cells grew to 80 % confluency, the primary mammary epithelial cells were trypsinized with 0.25 % trypsin plus 0.02 % EDTA and passaged. The pure mammary epithelial cells were isolated after 3 to 4 passages.

To confirm that the cells we isolated were pure mammary epithelial cells, cells plated on glass coverslips were washed twice with PBS and fixed in ice-cold methanol for 10 min. Cells were blocked with 10 % normal rabbit serum (ZLI-9025, ZSGB-BIO, Beijing, China) in PBS at room temperature for 1 h and then treated with cytokeratin 18 antibody (diluted 1:200 in PBS containing 10 % normal rabbit serum; sc-31700, Santa Cruz, CA, USA) at 4 °C overnight. Following three washes with PBS, cells were incubated with fluorescein isothiocyanate (FITC)-conjugated AffiniPure rabbit anti-goat IgG (diluted 1:50 in PBS containing 10 % normal rabbit serum; ZF-0314, ZSGB-BIO, Beijing, China) for 1 h at room temperature. To stain the nuclei, cells were treated with propidium iodide (Sigma-Aldrich, St Louis, MO, USA) for 10 min at room temperature, followed by three washes with PBS, and mounted with DABCO (Sigma-Aldrich, St Louis, MO, USA). Fluorescence was assessed by confocal microscopy (TCS SP2, Leica Microsystems, Wetzlar, GmbH, Germany).

To investigate if glucose has the ability to induce cell growth and lactose synthesis, mammary epithelial cells were plated at 6 × 10^4^ cells/well in 12-well culture plates. When cells grew to 80 % confluency, the medium was changed to DMEM with high glucose (25 mM, 11965–092, Life Technologies, Carlsbad, CA, USA) or without glucose (11966–025, Life Technologies, Carlsbad, CA, USA). After 24 h of treatment, cells in triplicate wells were collected for viability and proliferation analyses, and medium in triplicate wells was collected for lactose content analysis.

To detect the effects of different concentrations of glucose on cell growth and lactose synthesis, mammary epithelial cells were plated at 6 × 10^4^ cells/well in 12-well culture plates. When cells grew to 80 % confluency, the medium was changed to DMEM with different concentrations of glucose (ranging from 0 to 20 m*M*). After 12, 24, 36, and 48 h of treatment, cells in triplicate wells were collected for viability and proliferation analyses, and medium was collected for lactose content analysis.

To explore the molecular process by which glucose induces lactose synthesis, mammary epithelial cells were plated at 2 × 10^5^ cells/well in 6-well culture plates. When cells grew to 80 % confluency, the medium was changed to DMEM with 12 m*M* glucose or without glucose. After 24 h of treatment, cells in triplicate wells were collected for indicated assays.

### Cell viability and proliferation analyses

Cell viability and proliferation were assayed with the CASY-TT Analyzer System (Schärfe System GmbH, Reutlingen, Germany) as described previously [45]. Briefly, 100 μL mammary epithelial cell-suspension aliquots were transferred to a CASY cup containing 10 mL CASY ton, mixed by inverting three times, and placed in the CASY cell counter. All experiments were performed in triplicate.

### Lactose analysis

Lactose content in medium was measured with the Lactose/d-Glucose (Rapid) Assay kit (K-LACGAR, Megazyme, Ireland, UK) as described [46]. All experiments were performed in triplicate.

### qPCR

Total RNA was extracted from each well using TRIzol reagent (15596–026, Life Technologies, Carlsbad, CA, USA). M-MLV reverse transcriptase (28025–013, Life Technologies, Carlsbad, CA, USA) was used with oligo (dT) primers to perform first-strand synthesis. Quantitative real-time PCR was performed using mRNA-specific primers for *GLUT1*, *GLUT4*, *GLUT8*, *GLUT12*, *SLC35A2, SLC35B1, AKT1*, *HK2*, *α-LA*, and *β4GalT-I* (Table 1) in the Applied Biosystems 7300 Real-time PCR system (Applied Biosystems, NY, USA). Primers were designed using Primer Premier 5.0 (PREMIER Biosoft, Palo Alto, CA) with amplicon size more than 100 bp and limited 3’ G + C. Major parts of the primers were designed to span the junctions of 2 exons when possible to avoid amplification of genomic DNA. Prior to qPCR the primers were tested by a 20 μL PCR reaction using the same protocol described for qPCR and the PCR products were analyzed by gel electrophoresis on 2 % agarose gels (Additional file 1). The primers that presented a single band at the expected size were used for qPCR. The qPCR was performed in a 20-μL volume containing 10 μL of SYBR Premix Ex Taq II (RR820A, TaKaRa, China, Dalian), 0.4 μL ROX Reference Dye II (RR820A, TaKaRa, China, Dalian), 0.8 μL each of 10 μM forward and reverse primers, 2 μL cDNA and 6 μL DNase/RNase free water. Reactions were performed using the following conditions: 30 s of predenaturalization at 95 °C, followed by 40 cycles of 5 s denaturation at 95 °C and 34 s annealing and extension at 60 °C. The presence of a single PCR product was verified by the dissociation protocol as follows: 15 s at 95 °C, followed by 1 min at 65 °C, and 15 s at 95 °C. Each gene was amplified in a separated reaction and triplicate PCRs were performed on each sample of cDNA. Repeatability (intra-assay) and reproducibility (inter-assay) were assessed by computing the coefficient of variation under different conditions. The intra-assay variability was tested in triplicate in the same run. The inter-assay variability was evaluated in three independent runs, performed in different days. The intra-assay and inter-assay coefficients of variation were both less than 2 %. The comparative Ct method was used to analyze the relative changes of each gene [47]. The abundance of *β-ACTIN* was not significantly different between treatments, therefore, *β-ACTIN* was chosen for the purpose of normalization.

**Table 1:** Primers sequences used for qPCR

Gene symbol^1^	GenBank accession	Primer sequence (5′ to 3′)^2^	Melting temperature	Product size
SLC2A1	NM_174602.2	F: GACACTTGCCTTCTTTGCCA	58.97	164 bp
		R: AACCTAATGGAGCCTGACCC	59.08	
SLC2A4	NM_174604.1	F: TCATTCTTGGACGGTTCTTC	55.40	159 bp
		R: CTAGCACCTGGGCGATTA	56.08	
SLC2A8	NM_201528.1	F: CGGCTCAGAACCTGTGGAT	59.40	165 bp
		R: GGAGGATGCCTGTGACTACC	59.53	
SLC2A12	NM_001011683.2	F: ATTGTCATCGGCATTCTT	51.80	165 bp
		R: ATGTCCCTTCATCACCAG	53.43	
SLC35A2	NM_176640.2	F: GTGGTCCAGAATGCTTCCCTC	60.68	163 bp
		R: CCAGGTGCTTCACGTTACCC	60.95	
SLC35B1	NM_181338.2	F: GACCTGCTCCATCATCACCAC	60.75	126 bp
		R: AGACCGAGACCCAAGAACACC	61.71	
AKT1	NM_173986.2	F: TCATGCAGCACCGATTCTT	57.44	159 bp
		R: CTTGGTCAGGTGGCGTAAT	57.45	
HK2	XM_015473383.1	F: AAGATGCTGCCCACCTACG	59.78	123 bp
		R: TCGCTTCCCATTCCTCACA	58.63	
LALBA	NM_174378.2	F: AGACTTGAAGGGCTACGGA	57.62	177 bp
		R: TAGTTGCTTGAGTGAGGGTT	56.38	
B4GALT-I	NM_177512.2	F: TGCCCTGAGGAGTCCCC	59.92	151 bp
		R: GGCCACCTTGTGAGGAGAG	59.70	
ACTB	NM_173979.3	F: AGGACCTCTACGCCAACACG	61.87	249 bp
		R: TTTGCGGTGGACGATGGAG	60.38	

^1^SLC2A1 = glucose transporter 1(GLUT1); SLC2A4 = glucose transporter 4 (GLUT4); SLC2A8 = glucose transporter 8 (GLUT8); SLC2A12 = glucose transporter 12 (GLUT12); SLC35A2 = solute carrier family 35 member A2; SLC35B1 = solute carrier family 35 member B1; AKT1 = PKB alpha; HK2 = hexokinase 2; LALBA = α-lactalbumin (α-LA); B4GALT1 = β-1,4-galactosyltransferase 1 (β4GalT-I); ACTB = β-ACTIN

^2^F: forward; R: reverse

### Small interfering RNA (siRNA)-mediated gene knockdown

The siRNA against *AKT1* and scrambled siRNA (Scr) were chemically synthesized by GenePharma Co., Ltd, Shanghai, China. Gene knockdown was achieved by transient transfection with *AKT1* siRNA or scrambled siRNA as negative control using Lipofectamine 2000 Reagent (11668–019, Life Technologies, Carlsbad, CA, USA) as described previously [16]. Transfection efficiency was detected by fluorescence microscope (DM LB2, Leica Microsystems, Wetzlar, GmbH, Germany) after 6 h of transfection (Additional file 2). After 48 h of transfection, cells were extracted for the *AKT1* mRNA and protein expression detection.

To investigate the effect of *AKT1* knockdown on lactose synthesis in mammary epithelial cells, after 48 h of transfection, the medium was changed to DMEM with different concentrations of glucose (ranging from 0 to 20 m*M*). After 24 h of treatment, cells and medium in triplicate wells were collected for indicated assays.

### Western blot analysis

For western blot analysis, dairy cow mammary epithelial cells were washed twice with ice-cold PBS and lysed with RIPA buffer (P0013C, Beyotime Biotechnology, Shanghai, China) containing 0.5 m*M* PMSF (ST506, Beyotime Biotechnology, Shanghai, China),5 μg/mL aprotinin (A1153, Sigma-Aldrich, St Louis, MO, USA) and 5 μg/mL leupeptin (L2884, Sigma-Aldrich, St Louis, MO, USA ) on ice. The cell lysates were centrifuged at 17,800 × g for 10 min at 4 °C. Protein quantification was performed using the BCA Protein Assay kit (23227, Thermo Fisher Scientific Inc., Waltham, MA, USA). Equal amounts of protein (40 μg) were separated by 10 % SDS-PAGE and transferred onto nitrocellulose membranes. Membranes were blocked for 1 h with 5 % nonfat milk in Tris-buffered saline with 0.1 % Tween 20 (TBST) at room temperature and incubated overnight at 4 °C with primary antibodies specific to GLUT1 (1:200 dilution; sc-7903, Santa Cruz, CA, USA), SLC35A2 (1:200 dilution; sc-82032, Santa Cruz, CA, USA), SLC35B1 (1:200 dilution; sc-132864, Santa Cruz, CA, USA), HK2 (1:200 dilution; sc-6521, Santa Cruz, CA, USA), β4GalT-I (1:200 dilution; sc-22279, Santa Cruz, CA, USA), AKT1 (1:200 dilution; sc-1618, Santa Cruz, CA, USA), pAKT1 (1:1000 dilution, Abcam, Cambridge, MA, USA), and β-ACTIN (1:1000 dilution; 4970, Cell Signaling Technology, Beverly, MA, USA). The primary antibodies used were all diluted in 5 % BSA in TBST. The blots were then treated with horseradish peroxidase (HRP)–conjugated anti-goat IgG (1:500 dilution; ZB-2306, ZSGB-BIO, Beijing, China) or HRP-conjugated anti-rabbit IgG (1:500 dilution; ZB-2301, ZSGB-BIO, Beijing, China) for 1 h at room temperature. Proteins were visualized using the SignalFire ECL Reagent (6683, Cell Signaling Technology, Beverly, MA, USA). Three samples were assessed per time point or treatment. The protein bands were used for densitometric analysis using Image-Pro Plus 6.0 (Media Cybernetics, Inc., Warrendale, PA, USA) to obtain relative protein levels expressed as integrated density values. The integrated density, representing the mean density of pixels multiplied by the area was determined from equal sized rectangles drawn around the bands of interest minus the background to remove non-specific antibody staining. The protein levels were normalized to β-ACTIN.

### Statistical analysis

Experimental data were analyzed using GraphPad Prism 6 (GraphPad Software, La Jolla, CA). Quantitative data is presented as the mean ± SEM. A two-tailed unpaired *t*-test was used for two condition comparison. Two-way or one-way ANOVA with Bonferroni multiple comparison was used for multiple conditions comparison. *P* < 0.05 was considered to be statistically significant.

## Abbreviations

UDP, uridine diphosphate; β4GalT-I, β-1, 4-galactosyltransferase, α-LA: α-lactalbumin; PKB, the protein kinase B, also known as AKT; GLUT, glucose transporter; qPCR, quantitative real-time PCR; SLC35A2, solute carrier family 35 member A2; SLC35B1, solute carrier family 35 member B1; HK2, hexokinase 2; Scr, scrambled siRNA; FITC, fluorescein isothiocyanate.

## Additional files

Additional file 1: Agarose gel electrophoresis of the genes amplicons after qPCR. (DOCX 192 kb) Additional file 2: Transient transfection of mammary epithelial cells from dairy cows with AKT1 siRNA and scramble siRNA. (DOCX 337 kb)
