# Supplementary material for: Effects of glucose on lactose synthesis in mammary epithelial cells from dairy cow
Source: BMC Vet Res. 2016 May 26;12:81. doi: 10.1186/s12917-016-0704-x (PMC4880877; doi:10.1186/s12917-016-0704-x)

**Additional File 1:**

**Supplementary Figure1. Agarose gel electrophoresis of the genes amplicons after qPCR.** The primers were tested by a 20-μL PCR reaction using the same protocol described for qPCR and the PCR products were analyzed by gel electrophoresis on 2% agarose gels. M, DNA marker (DL500, Takara).


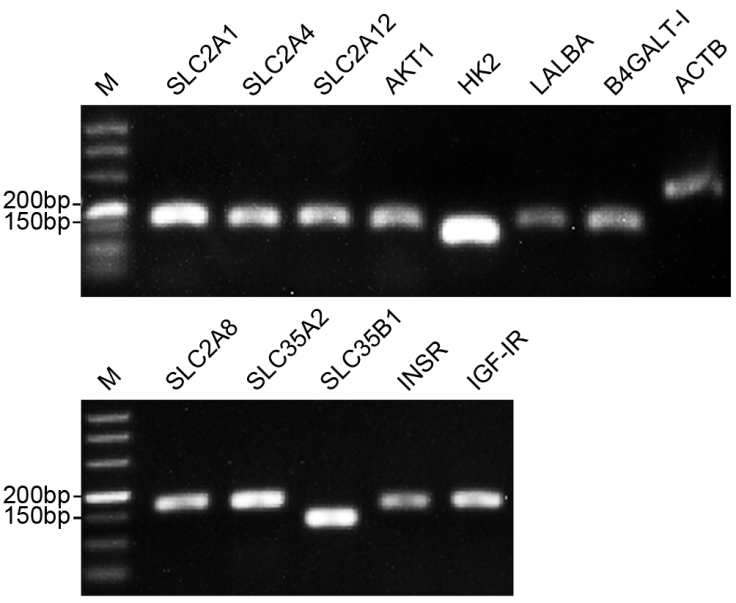

Supplement: Additional file 1: — Agarose gel electrophoresis of the genes amplicons after qPCR. (DOCX 192 kb) [file 12917_2016_704_MOESM1_ESM.docx]
