# Supplementary material for: Effects of glucose on lactose synthesis in mammary epithelial cells from dairy cow
Source: BMC Vet Res. 2016 May 26;12:81. doi: 10.1186/s12917-016-0704-x (PMC4880877; doi:10.1186/s12917-016-0704-x)

**Additional File 2:**

**Supplementary Figure 2. Transient transfection of mammary epithelial cells from dairy cows with *AKT1* siRNA and scrambled siRNA.** Uptake of the fluorescent-labeled AKT1 siRNA (A) and scramble siRNA (B) were assayed after 6 h of transfection. Fluorescent image of the transfected cells were taken by fluorescence microscope. Scale bar, 150 μm. This experiment was performed in triplicate.


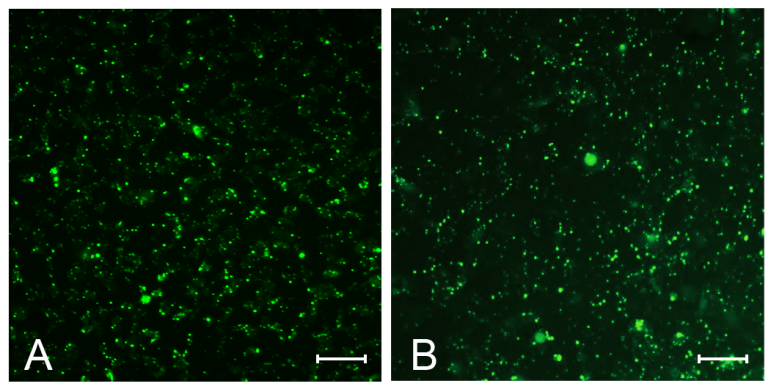

Supplement: Additional file 2: — Transient transfection of mammary epithelial cells from dairy cows with AKT1 siRNA and scramble siRNA. (DOCX 337 kb) [file 12917_2016_704_MOESM2_ESM.docx]
